# Universal lineshapes at the crossover between weak and strong critical coupling in Fano-resonant coupled oscillators

**DOI:** 10.1038/srep24592

**Published:** 2016-04-19

**Authors:** Simone Zanotto, Alessandro Tredicucci

**Affiliations:** 1 Istituto Nazionale di Ottica - CNR, Via Nello Carrara 1, 50019 Sesto Fiorentino (FI), Italy; 2 NEST, Istituto Nanoscienze - CNR, P.za S. Silvestro 12, 56127 Pisa, Italy; 3 Dipartimento di Fisica “E. Fermi”, Università di Pisa, Largo Pontecorvo 3, 56127 Pisa, Italy

## Abstract

In this article we discuss a model describing key features concerning the lineshapes and the coherent absorption conditions in Fano-resonant dissipative coupled oscillators. The model treats on the same footing the weak and strong coupling regimes, and includes the critical coupling concept, which is of great relevance in numerous applications; in addition, the role of asymmetry is thoroughly analyzed. Due to the wide generality of the model, which can be adapted to various frameworks like nanophotonics, plasmonics, and optomechanics, we envisage that the analytical formulas presented here will be crucial to effectively design devices and to interpret experimental results.

Among the resonance lineshapes, Fano lineshapes deserve a special interest since they first enabled to interpret atomic and molecular physical processes^1^, and subsequently the response of nanostructured systems like photonic crystals and plasmonic resonators^2^,^3^. In general, resonances may be absorptive, in the sense that part of the energy vehicled by the excitation field is converted into other forms. While absorption, in the sense of losses, is usually an undesired effect, in other frameworks it can be harnessed to enable functional operations like detection, wavelength conversion or quantum state transfer. Absorbing systems also exhibit a rich physics, as for instance that of coherent perfect absorption (CPA) which shares some key mathematical aspects with non-Hermitian quantum systems and parity-time symmetry breaking^4^,^5^,^6^,^7^. In addition, CPA and related concepts may open new avenues in the control of wave properties like polarization^8^ or in the processing of chaotic signals^9^.

In its original formulation, the theory of CPA is very general, as it relies on fundamental properties of the scattering matrices^10^; when dealing with specific systems, appropriate modeling tools are needed. However, due to the possibly complex nature of the systems under analysis, microscopical approaches are often time-consuming, and an analytical model would be an advantage both for the interpretation of experimental results and as a guide to target ab-initio simulations. In this paper we analyze the CPA in a two-oscillator coupled-mode model, which, thanks to its generality, can be applied to a number of emergent frameworks like photon- or plasmon-exciton coupled systems^11^,^12^,^13^,^14^,^15^,^16^,^17^, multiplasmon resonators^18^,^19^,^20^, coupled photonic cavities^21^,^22^, and optomechanic devices^23^. Throughout the present work we will model the resonances through classical amplitudes rather than by field operators. This does not prevent the application of the model to systems which have a proper quantum nature, like the two-level system (or the collection of two-level systems) coupled to a cavity. Indeed, for those systems the Heisenberg-Langevin approach with a mean-field approximation leads to a set of equations which, in the weak-excitation limit, are a special case of the formulas given in the following. Our observations, however, advance the works published so far, since to our knowledge there is no systematic analysis of the simultaneous presence of a non-resonant background and two coupled resonances having similar lifetimes, arranged in a two-port configuration. For instance, ref. 12 deals with two coupled resonances not interacting with a non-resonant background; in ref. 20 instead, the analysis is focused on the interaction between a broad resonance and a narrow one, where the first effectively acts similarly to a continuum non-resonant background.

The key effect we would like to highlight is the emergence of certain features which generalize what is commonly understood as Fano lineshape. In detail, it will be shown that the Fano transmittance and reflectance resonances typical of an uncoupled symmetric resonator are inherited by the two-coupled-resonator system, possibly in the presence of asymmetry. Furthermore, it turns out that absorption lineshapes are described by another, universal lineshape, depending on few, physically meaningful parameters. A check of the model validity is finally provided, based on the concept of strong light-matter coupling in a realistic resonant metasurface embedding intersubband-active quantum wells.

## Description and Discussion of the Model

**Figure 1:**
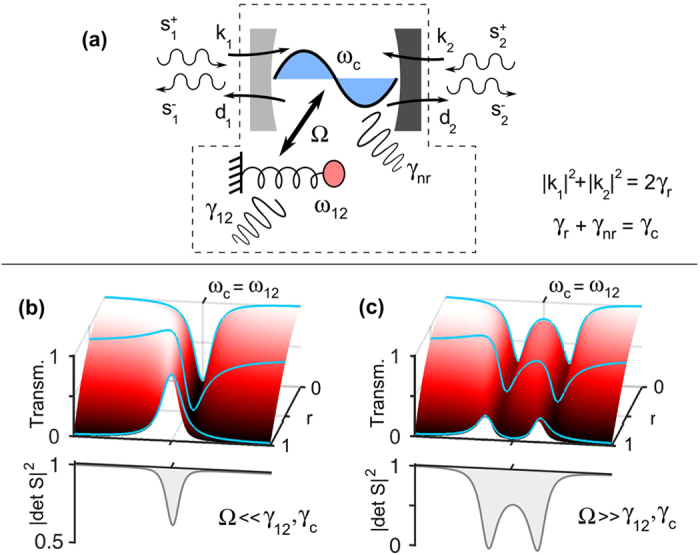
Panel (**a**): sketch of the coupled oscillator model analyzed in the article. Panels (**b**,**c**): spectral lineshapes of the weakly (**b**) and strongly coupled (**c**) system. The transmittance lineshape can be tuned through a parameter (*r*, see text), and is inherited from the weakly to the strongly coupled case. The S-matrix determinant, instead, is given by a universal function, independent of the trasmittance (and reflectance) lineshapes. Parameter values are *γ*_*r*_ = *γ*_12_, *ω*_*c*_ = *ω*_12_ = 50*γ*_*r*_, *γ*_*nr*_ = 0. In case (**b**) the ratio Ω/*γ*_*r*_ equals to 0.3, while in case (**c**) it equals to 2.7.

The model under consideration is schematized in Fig. 1(a). A resonant cavity at frequency *ω*_*c*_ is coupled to a second resonant degree of freedom, here represented as a spring-mass resonator at *ω*_12_, through a coupling coefficient Ω. From now on, the second oscillator will be referred to as “matter” resonator, since a prototypical situation would be that of a two-level system (atom, exciton) treated under the semiclassical approximation. However, another important situation could be that of a subradiant (“dark”) mode in a plasmonic system, and the notation would assume a different meaning. The cavity resonator radiates into, and is excited from, two radiative scattering channels through two ports, with couplings *d*_1,2_ and *κ*_1,2_. *γ*_12_ describes an internal loss mechanism of the matter resonator, while *γ*_*nr*_ describes a *non-radiative* and *non-resonant* cavity loss mechanism. In the photonic framework, *γ*_*nr*_ may represent losses such as roughness scattering or dissipation in a metal component.

The dynamics of the system is described by


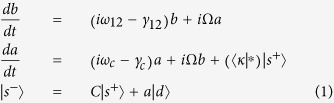


where *a* and *b* are the amplitudes of, respectively, the cavity and the matter resonators. Here, 
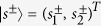
 are column vectors describing the amplitudes of ingoing and outgoing scattered waves. Similarly, 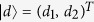 and 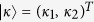. In this notation, the transformation 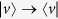 means transposition and complex conjugation, while 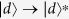 means only complex conjugation. *C* is a scattering matrix which describes the background, non resonant process.

According to the above equations, the free evolution of the cavity resonator occurs with a damping rate *γ*_*c*_, which describes its total losses, radiative plus nonradiative. Indeed, it can be decomposed as *γ*_*c*_ = *γ*_*r*_ + *γ*_*nr*_, where the second term has the meaning described above, while the first (the purely radiative damping rate) must satisfy 2*γ*_*r*_ = 〈*d*|*d*〉. This constraint can be derived by imposing instantaneous energy conservation to Eqs. 1, having interpreted |*a*|^2^ and |*b*|^2^ as oscillator energies, and 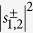 as energy fluxes to/from the system. Actually, energy conservation and time-reversal symmetry constraints also require |*κ*〉 = |*d*〉 and *C*|*d*〉* = −|*d*〉, as already observed for the single-oscillator case^22^.

The linear response of the system is fully described by its scattering matrix *S*, which links the amplitudes of ingoing and outgoing waves through 
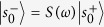
. Direct integration of Eqs. 1 yields


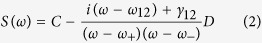


where 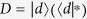. The explicit expression of the poles *ω*_±_ will be given in the following, while matrices *C* and *D* read


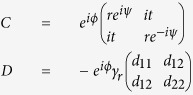


where


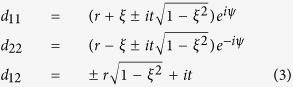


Matrix *C* is the most general two-by-two unitary and symmetric complex matrix, enforcing energy conservation and reciprocity of the direct scattering process. Apart from the phase factors, it depends on the single parameter *r* ∈ [0, 1] (being 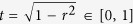), which describes the off-resonant reflection amplitude. Matrix *D* involves an additional parameter *ξ*, which describes the asymmetry of the decay into the scattering channels. This parameter, constrained in the interval [−1, 1], is connected with the coupling coefficients through the relation 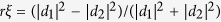. This link between *d*_1,2_ and *r*, already outlined for single-mode optical resonators^24^, is here generalized to the two-oscillator model.

In this model, if the cavity is decoupled from the matter resonator (Ω = 0) and there are zero non-resonant losses (*γ*_*nr*_ = 0), the results of ref. 24 are recovered. Considering the transmission spectra, Fano lineshapes are obtained; similar curves are observed in reflection. Similar lineshapes, although less contrasted, are observed under the weak cavity-matter coupling regime, i.e., when the coupling Ω is finite but smaller than *γ*_12_ and *γ*_*c*_. As shown in Fig. 1(b), the lineshapes sweep from a Lorentzian to an inverted Lorentzian according to the value of the parameter *r*. Here, we assumed *ξ* = 0, while it can be shown that *ξ* ≠ 0 leads to a further decrased lineshape contrast. Consider now the spectra in Fig. 1(c), obtained under the condition that Ω is larger than *γ*_12_ and *γ*_*c*_. The two resonators are strongly coupled, and the spectral feature is doubled, following the peak, dip, or asymmetric shape of the weakly coupled system. This unique behaviour (“*lineshape inheritance*”), which was already observed experimentally and justified heuristically^25^, is now grounded on a basic theoretical model, and can be extended to all systems which can be described by Eq. (1).

In the above, the focus was on transmittance and reflectance. While in an experiment these are the most easily accessible quantities, a more meaningful probe of a driven linear system would rather be the absorption, since it is directly connected to the excitation of the resonant degrees of freedom. Here, the model under consideration presents two scattering channels, and should be analyzed in view of the coherent absorption theory for asymmetric two-port systems^10^,^26^. A key quantity is the *S*-matrix determinant, which, following Eq. 2, reads


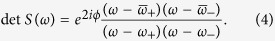


The zeroes, which are connected to the coherent perfect absorption (CPA) condition, explicitly read


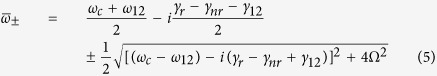


while the poles are obtained from the expression above by replacing *γ*_*r*_ with −*γ*_*r*_. The essential feature of Eq. 4 is that it does depend *neither* on *r nor* on *ξ*: the lineshape-governing factors do not influence the *S*-matrix determinant. This behaviour is represented in the graphs on the lower side of Fig. 1(b,c).

**Figure 2:**
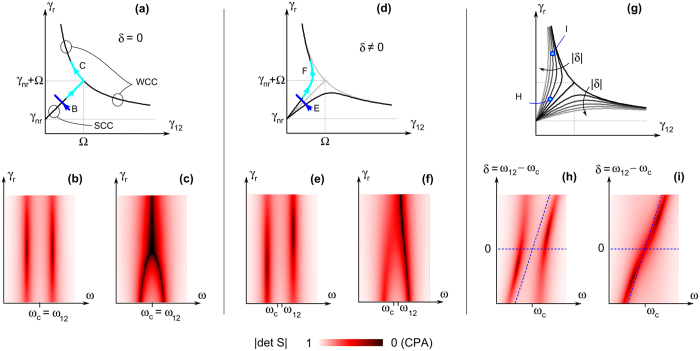
Coherent perfect absorption (CPA) in the dissipative coupled oscillator model. The panels on the top represent the phase diagram of the system: on the highlighted curves (criticality conditions), CPA occurs. Moving from degenerate (*δ* = 0) to non-degenerate (*δ* ≠ 0) cases, a rearrangement of CPA conditions is observed. The spectral behaviour is encoded in the color maps on the bottom, which represent the S-matrix determinant. The position of critical curves and of the S-matrix determinant spectral lineshapes are independent of the specific Fano lineshape occurring on the transmission/reflection spectra.

What is more, the above expressions imply that the CPA condition itself is not influenced by the lineshape-governing factors. Looking for a closed form for the CPA condition, one indeed obtains


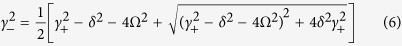


where *γ*_−_ = *γ*_*r*_ − *γ*_*nr*_ − *γ*_12_, *γ*_+_ = *γ*_*r*_ − *γ*_*nr*_ + *γ*_12_, and *δ* = *ω*_12_ − *ω*_*c*_. Eq. 6 unifies and generalizes the weak and strong critical coupling concepts, which was introduced in ref. 27 for a symmetric (*ξ* = 0) and degenerate (*ω*_*c*_ = *ω*_12_) coupled resonator system. These concepts are recalled in Fig. 2(a–c): on the (*γ*_12_, *γ*_*r*_) plane the phase diagram of the degenerate system consists of well separated weak critical coupling (WCC) and strong critical coupling (SCC) curves. For parameters in the lower-left part of the phase diagram, the spectrum |det*S*(*ω*)| has a double-dip feature, with zeroes (i.e., CPA) when the SCC curve is intersected (see path B and the corresponding spectra in panel (b)). Exploring path C, instead, the coalescence of the two CPA zeros into a single one is revealed, i.e., the crossover between SCC and WCC through an exceptional point is observed (panel (c)).

Consider now the case *δ* ≠ 0. As depicted in Fig. 2(d), the curve which was representing the SCC condition splits up into two branches, which merge continuously into the former WCC curves. Meanwhile, the |det*S*| spectra change accordingly, as it can be observed from panels (e) and (f) which correspond to the paths E and F identified in panel (d). In the former SCC region, the determinant spectrum has still a double-dip, but CPA never occurs simultaneously for two frequencies. When the transition between the former SCC and WCC region is explored, the coalescence of |det*S*| minima still occurs, but only the higher frequency dip is a CPA. The effect of a continuous sweep of the detuning *δ* is finally analyzed in Fig. 2(g–i). Suppose that the system is close to, but not exactly on, the curve representing SCC (point H). Its spectrum, if *δ* = 0, has two dips which do not reach zero, as highlighted by the dashed horizontal line in (h); if *δ* is tuned, a CPA occurs either on the lower or on the higher frequency resonance of the coupled system. Suppose instead that the system is close to the WCC at *δ* = 0 (point I). Again, by a proper tuning of *δ* the system can be brought to CPA, but now with a single isolated spectral feature.

In essence, the detuning between the individual resonators which enter the coupled system has a twofold role. On one hand, it weakens the distinction between strong and weak critical coupling regimes, as when *δ* ≠ 0 the curves describing CPA on the phase diagram are smooth and do not exhibit any exceptional point. On the other hand, a proper tuning of *δ* can help a system to reach CPA, without any need to act either on the coupling Ω or on the damping rates *γ*. All these observations apply irrespectively of the asymmetry degree of the system and of the specific Fano lineshape that the system would show in reflection or in transmission, leading to a universal behaviour of coupled dissipative resonators.

## Numerical Validation of the Model on a Realistic System

To gain confidence in the theory detailed above, in the following we will analyze a resonant metasurface embedding semiconductor quantum wells (QWs). Such device implements a prototypical system in which weak and strong coupling have been observed, and where they can be harnessed to develop efficient mid-infrared and terahertz light sources, as well as functional optical components^28^,^29^.

**Figure 3:**
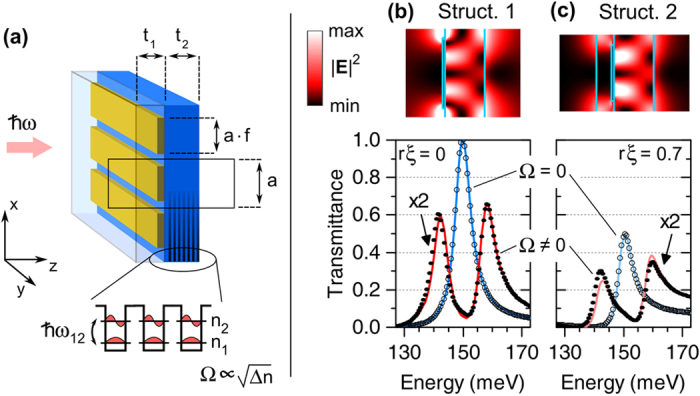
Panel (**a**), schematic of a resonant metasurface embedding quantum wells, which implement a prototype of strongly and critically coupled oscillators with asymmetry. Panels (**b**,**c**), resonant field and spectral transmittance of two structures which differ by the value of *t*_1_. These resonators are described by different asymmetry parameters, resulting in differently contrasted lineshapes. The calculations from a rigorous electromagnetic solver (dots) are faithfully reproduced by the coupled-mode model (red and blue lines).

A schematic of the structure is sketched in Fig. 3(a). It consists of a heterostructure of 60 equispaced GaAs/Al_0.33_Ga_0.67_As MQW, with well/barrier thicknesses 6.8/20 nm resulting in an intersubband transition frequency 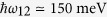
28. The membrane has thickness *t*_2_ = 1.3 *μ*m, and is periodically patterned with thin (50 nm) gold stripes, whose spacing is *a* and filling fraction is *f*. A high-index coating (*ε* = 10) coating with thickness *t*_1_ completes the layer stack. A fully vectorial electromagnetic modeling of the structure is performed through rigorous coupled wave analysis (RCWA). The permittivity of gold is assumed to be *ε*_Au_ = 4000 + 300*i*, that of GaAs *ε*_GaAs_ = 10, while the MQW response follows a Lorentz oscillator model (see refs 25,30 for details; the key parameters in this model are the resonance frequency *ω*_12_, the damping rate *γ*_12_ and the subband surface charge difference Δ*n*, which is eventually proportional to the oscillator strength). Suppose, at first, that the response of the MQW is turned off (Δ*n* = 0). The device exhibits an isolated photonic resonance at *ω*_*c*_ ≃ 150 meV, depending on the specific values of *a*, *f*, and *t*_1_. An analysis of the transmittance lineshape, following Eq. 2, revealed that the device acts as a symmetric resonator (i.e., *ξ* = 0), unless *t*_1_ ≠ 0. Indeed, if *t*_1_ = 0, the transmission lineshape is a fully-contrasted Lorentzian, which can only be reproduced with the coupled-mode model by setting *ξ* = 0. If instead a finite value of *t*_1_ is chosen, the transmission lineshape has a contrast smaller than one, which can be only reproduced by setting *ξ* ≠ 0. For the present structure the lineshapes turned out to be almost pure upward Lorentzians, hence the non-resonant scattering parameter *r* is expected to be very close to unity.

Aiming at understanding the role of asymmetry in strongly-coupled dissipative resonators, we studied two structures: one with *t*_1_ = 0, and the other with *t*_1_ = 0.5 *μ*m. By tuning *a* and *f* both structures have the resonance frequency close to 150 meV (see Table 1). The numerically calculated transmission spectra corresponding to these devices are reported as empty dots in Fig. 3(b,c), while the fit resulting from Eq. 2 is represented by the blue lines. From Table 1 it can be noticed that both structures have similar radiative lifetimes *γ*_*r*_ and non-resonant reflection *r*, while they have different asymmetry parameter *ξ* (in the case of Struct. 1, the precise fitting procedure led to a very small, but finite, value of *ξ*). Meanwhile, as it can be observed from the color maps in Fig. 3, the resonantly excited intracavity field profile is almost similar for both devices, except for a slightly better confinement for structure 2 (see also the overlap factors in Table 1, defined as 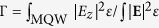, where MQW stands for the quantum well region.).

The predictive value of the coupled-mode model is now checked by allowing the quantum wells to be active as a two-level system coupled with the cavity, which implies Ω ≠ 0 in the sketch of Fig. 1. The coupling constant can indeed be determined ab-initio by identifying it with the vacuum Rabi frequency, which reads 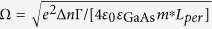, where *ε*_GaAs_ is the well material permittivity, m* = 0.067*m*_0_ is the conduction subband effective mass, and *L*_*per*_ is the QW period thickness. With Δ*n* = 5 × 10^11^ cm^−2^, the coupling is 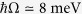. We also assumed that the intersubband transition frequency and decay rate closely match those of the photonic cavity: 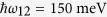, 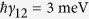. These numbers, together with the other parameters extracted from the empty cavity spectra, are then plugged into Eq. 2, resulting in the transmittance curves reported as red lines in Fig. 3(b,c). Such double-peaked spectra should be compared with the dotted traces, which were obtained through the RCWA simulation of the photonic crystal loaded with the active MQW. Although the peak values of numerical and analytical spectra are slightly different (which is likely to be attributed to higher-order photonic resonances, not included in the present model), the overall agreement is very good. This witnesses that the analytical expressions provided by the coupled-mode model can quantitatively predict the response of an asymmetric two-port photonic system strongly coupled with a matter excitation.

To conclude the analysis of the asymmetric and detuned coupled oscillator model, we study the response of the asymmetric intersubband polariton samples to a double-sided optical excitation. Absorption of optical radiation from such systems is indeed a meaningful figure of merit, for instance for detectors and for other devices where an efficient pumping of the polariton population is needed.

The key quantity is hence the joint absorption *A*_j;±_, as defined in ref. 26. Starting from Eq. 2, algebraic manipulations lead to


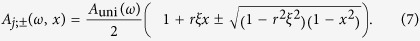


Here, 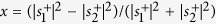 is the asymmetry between the input intensities on the two ports, and the sign ± represents the minimum and maximum absorption achievable by acting on the relative phase of the input beams. Notice that *A*_j_ as a function of *x* correctly shows the peculiar elliptical behaviour of coherent absorption^26^. The significant feature standing out from Eq. 7 is that the ellipse is described by the sole function in parentheses, which factors out from the universal spectral lineshape function 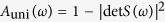. Hence, it is only the function in parentheses, which we label *f*_±_(*r*, *ξ*, *x*), that summarizes the effect of asymmetry on coherent absorption. Instead, the universal lineshape function is independent on both *r* and *ξ*. This observation goes beyond what has been stated in the first part of the paper, when discussing Eq. 6: it is not only the CPA condition which does not involve *r* and *ξ*, but the joint absorption lineshape in its fullness.

**Figure 4:**
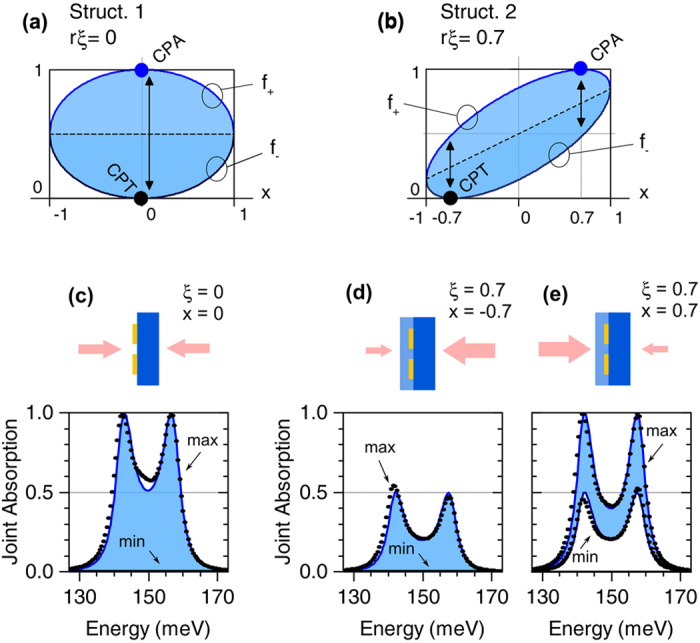
Coherent absorption universal factor *f*_±_ (see the comments to Eq. 7 for details) for a coupled-resonator system which behaves symmetrically (**a**) or asymmetrically (**b**). Minimum and maximum joint absorption for structure 1 (**c**) and structure 2 (**d**,**e**). Both structures show coherent perfect absorption and transparency (CPA and CPT), but in the latter those are observed at different *x*-values, i.e., for different states of external excitation, due to the asymmetric behaviour of the cavity.

In Fig. 4(a,b) we plot *f*_±_(*x*), where the choices *rξ* = 0 and *rξ* = 0.7 recall the actual values of structures 1 and 2 (see Table 1). The function *f*_−_ is zero for *x* = −*rξ*, which means that the system can always exhibit coherent perfect transparency (CPT) provided that the excitation intensities properly match the intrinsic asymmetry of the photonic resonance. For what concerns CPA, instead, necessary and sufficient condition is that *A*_uni_ = 1 (and hence |det*S*| = 0), *and f*_+_ = 1, i.e., *x* = *rξ*. In other words, there are two independent requirements: the first on damping rates, detuning, and coupling coefficient (Eq. 6), the second on the symmetry.

A numerical test is then proposed in Fig. 4(c–e), where *A*_j_ calculated numerically for structures 1 and 2 is compared with the prediction of Eq. 7. No further parameters in addition to those already given in Table 1 are involved, neither in the numerical nor in the analytical calculation. Since *δ* = 0 and *γ*_*r*_ = *γ*_12_, both samples are in strong critical coupling and CPA is expected. Indeed, when the proper *x*-value is chosen, *A*_j_ reaches 1 in a double-peaked fashion for both samples (Fig. 4(c,e)). Since structure 1 behaves as an optically symmetric resonator (*ξ* = 0), CPA and CPT occur simultaneously for *x* = 0; in structure 2, instead, CPA and CPT occur for opposite values *ξ* = ±0.7, consistently with Eq. 7.

## Conclusions and Perspectives

In summary, we studied the absorption lineshapes occurring at the transition between weak and strong critical coupling regimes for a system consisting of two coupled detuned resonators, one of which is radiatively coupled with the exterior in an asymmetric fashion. From this model a peculiar fingerprint in the absorption spectra stands out: a universal lineshape, independent of the asymmetry degree, which instead rules the Fano lineshapes observed in transmittance or in reflectance. Similarly, the coherent perfect absorption (CPA) condition results to be independent of the lineshape-governing factors. Rather, it turns out that the CPA phase diagram is significantly affected by the oscillator’s detuning, whose effect is to weaken the distinction between weak and strong (critical) coupling regimes. Being the present model of wide generality, it is of significance in the development of many active research topics like nanophotonics, plasmonics or optomechanics, where the modeling of a complex system would draw advantage from analytical expressions depending on few parameters of direct interpretation.

## Additional Information

**Data Availability**: The computational software employed in this work (PPML) can be found online at http://www.mathworks.com/matlabcentral/fileexchange/55401-ppml-periodically-patterned-multi-layer.

**How to cite this article**: Zanotto, S. and Tredicucci, A. Universal lineshapes at the crossover between weak and strong critical coupling in Fano-resonant coupled oscillators. *Sci. Rep.* **6**, 24592; doi: 10.1038/srep24592 (2016).

## Figures and Tables

**Table 1:** Geometric data and coupled-mode model parameters for the structures analyzed in Figs 3 and 4.

Struct.	*t*_1_ [*μ*m]	*a*[*μ*m]	*f*	*ω*_*c*_[meV]	*γ*_*r*_[meV]	*r*	*ξ*	Γ
**1**	0	3.70	0.80	149.60	3.25	0.996	0.001	0.47
**2**	0.5	3.20	0.73	149.70	2.77	0.981	0.72	0.56
