# Corrigendum: Universal lineshapes at the crossover between weak and strong critical coupling in Fano-resonant coupled oscillators

**DOI:** 10.1038/srep27046

**Published:** 2016-06-02

**Authors:** Simone Zanotto, Alessandro Tredicucci

Scientific Reports 6: Article number: 24592;10.1038/srep24592 Published online: 04192016; Updated: 06022016

The Acknowledgements section in this Article is incomplete.

“The authors gratefully acknowledge Raffaele Colombelli for precious suggestions and insights on the application of coupled-mode models to strongly-coupled systems”.

should read:

“The authors gratefully acknowledge Raffaele Colombelli for precious suggestions and insights on the application of coupled-mode models to strongly-coupled systems. This work was in part supported by the European Research Council through the Advanced Grant SOULMAN (ERC-FP7-321122)”.

